# New method for sequestration of silver nanoparticles in aqueous media: in route toward municipal wastewater

**DOI:** 10.1186/s13065-016-0198-4

**Published:** 2016-08-26

**Authors:** Marie-Laine Roy, Christian Gagnon, Jonathan Gagnon

**Affiliations:** 1 Département de Biologie, chimie et géographie, Université du Québec à Rimouski, 300 allée des Ursulines, Rimouski, QC G5L 3A1 Canada; 2 Centre Saint-Laurent, Environment Canada, 105 McGill st., 7th floor, Montreal, QC H2Y 2E7 Canada

**Keywords:** Ag NP, Supported polysaccharide, Silica, Removal, Wastewater, Silver sulfide, Organic matter

## Abstract

**Background:**

Nanomaterials are widely used in industry for their specific properties. Silver nanoparticles (Ag NPs) are largely used in several consumer products notably for their antibacterial properties and will likely be found in wastewater, then in the receiving environment. The development of a product capable to sequestrate those released contaminants is needed. Under environmental conditions, the biopolymer chitosan can generally coordinate the cationic metals. Ag NPs present unique properties due to their high surface/mass ratio which are promising for their sequestration.

**Results:**

The immobilization of chitosan on functionalized silica assisted by microwaves gives a sequestering agent of silver while being easily recoverable. The IR spectrum confirmed the immobilization of *N*,*N*–dimethylchitosan (DMC) on silica core. The immobilized DMC gave a percentage of sequestration of Ag NPs (120 µg L^−1^) in nanopure water of 84.2 % in 4 h. The sequestration efficiency was largely dependent of temperature. By addition of hydrosulfide ions, the percentage of sequestration increased up to 100 %. The immobilized DMC recovered a large portion of silver regardless the speciation (Ag NP or Ag^+^). In wastewater, the immobilized DMC sequestered less Ag NPs (51.7 % in 97 % wastewater). The presence of anionic (sodium dodecyl sulfate and sodium *N*–lauroylsarcosinate) and non-ionic surfactants (cetyl alcohol) increased the hydrophobicity of Ag NPs and decreased the percentage of sequestration.

**Conclusions:**

The immobilized DMC is a promising tool for sequestrating such emerging pollutant at low concentrations in a large volume of sample that would allow the characterization of concentrated Ag NPs by transmission electron microscopy. The efficiency of the support to sequestrate would likely be influenced by the chemical environment of the Ag NP solution.

**Electronic supplementary material:**

The online version of this article (doi:10.1186/s13065-016-0198-4) contains supplementary material, which is available to authorized users.

## Background

Nanomaterials are widely used in industry for their specific properties. A nanoparticle is defined as a particle possessing at least two dimensions measuring between 1 and 100 nm [1, 2]. In recent years, silver nanoparticles (Ag NPs) have been widely studied since they have a high surface/mass ratio that confers a higher reactivity. They are used in catalysis and for their antimicrobial properties in many areas of applications including consumer products and textiles [1–4]. In 2012, approximately 55 tons of Ag NPs were produced and used [5]. The majority of Ag NPs in consumer products will be likely found in municipal wastewater treatment plants and exposure to aquatic organisms could result in different toxicological effects [6]. The development of new sequestration techniques is therefore important tools for their removal [2].

Chitosan represents a rare example of cationic biopolymer that is mainly extracted from crustacean exoskeletons. This aminopolysaccharide is known as coagulant and flocculent [7] and for its capacity to bind transition metals. The alcohol and amino groups in raw chitosan allow the chelation of transition metals. At neutral pH, cationic metals are coordinated by unbounded electrons of nitrogen atoms [4, 8]. Applications of chitosan are limited by its insolubility in aqueous solutions and organic solvents. The protonation of amino groups lead to the solubilization of chitosan in diluted acid conditions. However, its sorption capacity [4] and utilization in wastewater treatment [9] are limited. Ag NP recovery methods have been developed including cloud point extraction with Triton X-114 [10] and activated carbon [11]. These methods work for high concentrations of Ag NPs only.

Silica is a widely used support for chromatography and for supported reagents and catalysts [12]. Silica with silanol groups on the surface and a large surface area allow coupling with many molecules including polymers [8, 12]. The immobilization of polymers on silica can be used for a variety of applications such as biosensors and drug delivery, for instance [13]. The use of polymers in the catalytic reactions of chemicals and biological processes is growing. Supported polymers offer opportunities in the production of chemical and new intermediates [14]. Supported polymers are been used in various combinatorial chemicals, in the research for new drugs, in the oil refinery and in catalysis and biosynthesis [14, 15]. Supported polysaccharides allow the formation of support with high surface for sorption where some polysaccharides are used to immobilize various molecules such as enzymes. Some studies have been realized to immobilize chitosan on a support made of silica gel [16]. Immobilized chitosan can bound copper ions [9] or acted as affinity support for the adsorption of proteins [17]. These syntheses imply more than three steps that necessitate several days and require the removal of starting compounds. Moreover, concentrations of heavy metal ions were as high as the order of milligram per liter. The microwave-assisted heating is a technique with many advantages including the ability to accelerate chemical reactions and to achieve higher heating rates and better reaction yields [18, 19].

Therein, we report the preparation of immobilized chitosan derivative on modified silica and the assessment of potential sequestration of Ag NPs in municipal wastewater. In this work, the removal capacity of this sequestration was then studied against two other silver species (ionic silver and Ag_2_S NPs).

## Results and discussion

### Formation of immobilized *N*,*N*–dimethylchitosan (DMC) on modified silica

**Scheme 1:**
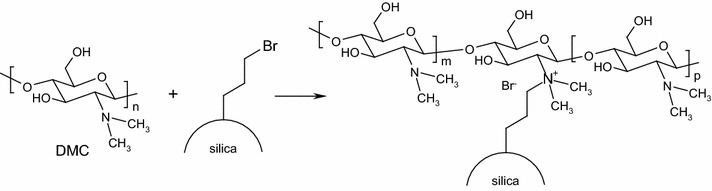
Immobilization of DMC on modified silica

The immobilization of DMC on the modified silica is summarized in Scheme 1. The alkyl halide of modified silica reacts with tertiary amine of DMC in a one-step process using microwave. Some tertiary amine groups are converted into quaternary ammonium allowing to chemically bound DMC onto silica propyl bromide. The reaction between DMC and modified silica lead to an insoluble product even in the protonated form whereas the free protonated DMC is soluble under acidic conditions. The immobilized DMC was washed with a solution of acetic acid to remove unbounded DMC. The supported DMC was then characterized by IR and Raman spectroscopy (see Additional file 1: Figures S1 and S2).

**Fig. 1:**
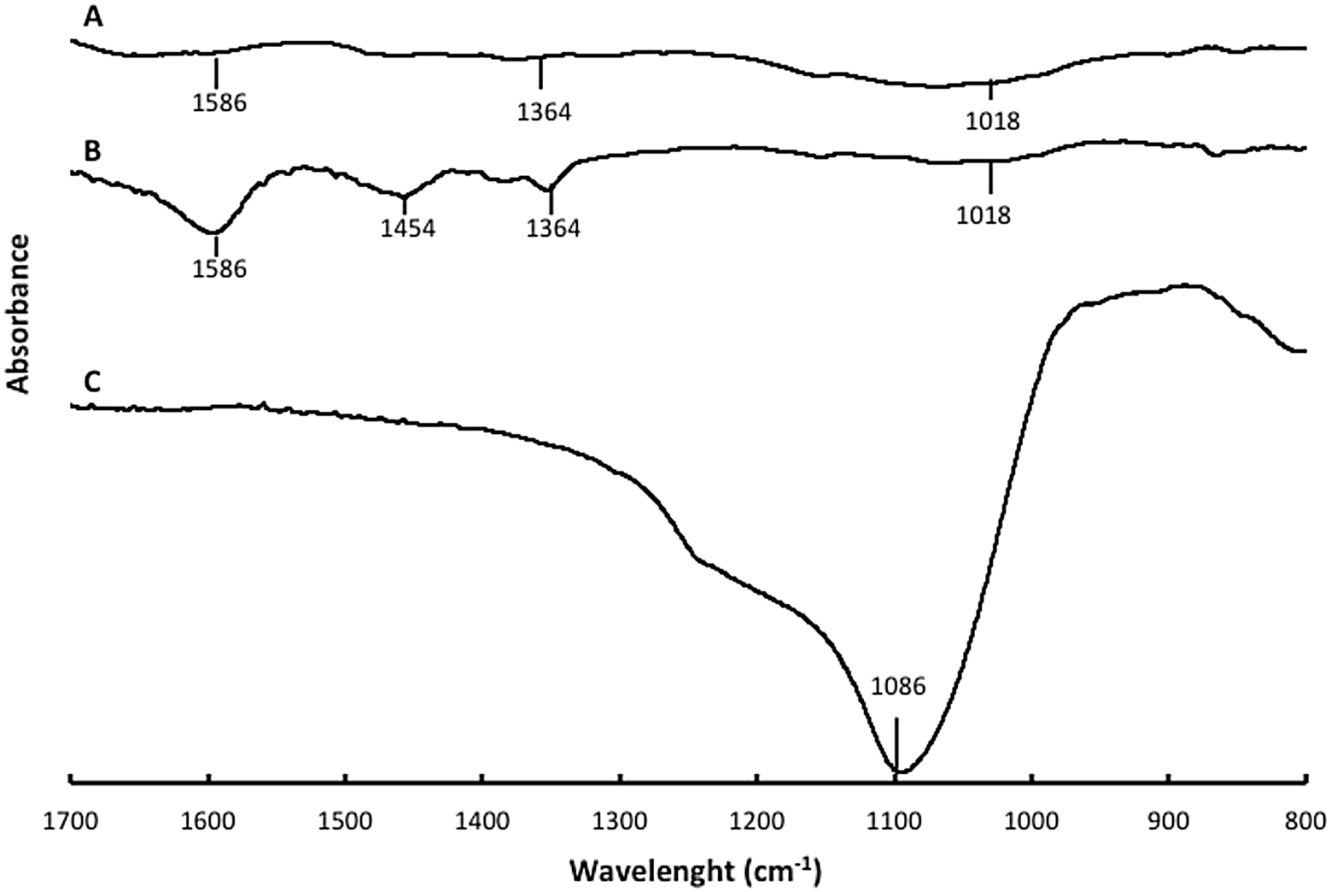
Infrared spectra in the 800–1700 cm^−1^ region of *A* immobilized DMC after washing; *B* DMC; *C* silica propyl bromide

In Fig. 1, the IR spectrum of silica propyl bromide shows a broad Si–O stretching at 1086 cm^−1^. The IR spectrum of DMC shows bands at 3423, 2869, 1586, 1455, 1364 and 1019 cm^−1^ representing OH stretching, CH vibrations, CH_2_ deformation, CH_3_ deformations, C-N stretching and C-O stretching, respectively (Additional file 1: Figure S1). The IR spectra of immobilized DMC after washing and those of free DMC are similar but the relative intensity of bands is different. The intensity of OH, C–O, C–N stretching are higher for immobilized DMC whereas the CH_2_ deformation band of immobilized polymer is lower. Considering that unbounded DMC was washed out, these bands indicate that DMC was fixed on the modified silica. These differences in IR spectroscopy indicate that the polymer is immobilized on silica and its surface is covered by DMC.

**Fig. 2:**
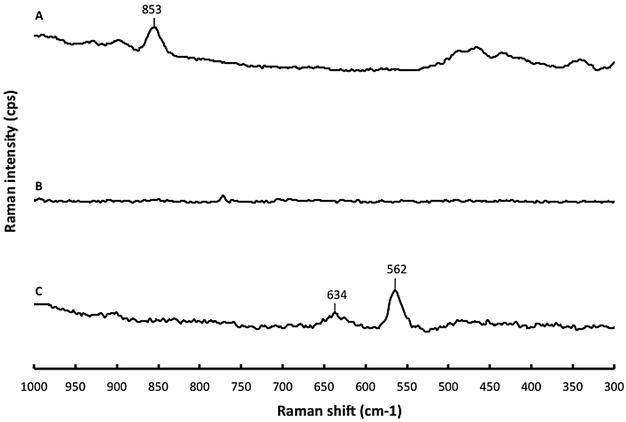
Raman spectra of *A* immobilized DMC after washing; *B* DMC; *C* silica propyl bromide

According to the literature [20], the C–Br stretching of bromoalkane compounds absorb in Raman at 645–635 and 565–555 cm^−1^ (general stretching zone). In Fig. 2, the Raman spectrum of the silica propyl bromide shows C–Br stretching at 634 and 562 cm^−1^. These vibrational bands disappeared after the immobilization of DMC. The disappearance of these bands in immobilized DMC spectrum indicates that DMC was bound to silica. The comparison of Raman spectra of immobilized DMC, free DMC and modified silica shows a new vibrational band at 853 cm^−1^ for immobilized DMC.

With the DMC/silica ratio used during the reaction, the nitrogen/carbon ratio of supports was quite constant within a variation of 5 %, a small decrease is observed for polymer/silica ratio of 2–5 (Additional file 1: Figure S3). The nitrogen percentage increases until a DMC/silica ratio of 1 and after is relatively constant as well demonstrating that immobilization of DMC on silica is saturated.

### Sequestration of silver nanoparticles

It is possible to qualitatively verify the sequestration of a solution of Ag NPs (120 µg L^−1^) by comparing UV–visible spectra before and after sequestration (Additional file 1: Figure S4). The intensity of the absorption band of citrate-coated Ag NPs at 400 nm decreases after sequestration that was attributed to the reduction of Ag NP concentration.

The ICP-MS analyses of the supernatant and immobilized DMC were carried out to verify the mass balance of silver content. Different sequestration parameters were evaluated whose influence of sequestration such as time, temperature and different forms of silver that can be found in the waters. These results are presented in Table 1. Table 1 (lines 1–3) shows the percentage of sequestration after 0.5, 2 and 4 h of Ag NPs in nanopure water. During the first 30 min, the support sequestrated a large proportion (59.9 %) of Ag NPs. After that the percentage of sequestration increased with time, but more slightly between 2 and 4 h to reach around 80 %. For the lower amount of ionic silver (1.34 mg L^−1^; line 6), the immobilized DMC recovered totally the metal. At higher concentration (4.25 mg L^−1^; line 7), the immobilized DMC sequestrated a lower proportion of ionic silver (84.2 %) since there must be probable saturation of the immobilized DMC. The maximum sorption capacity of the immobilized DMC at those concentrations was 10.1 µg g^−1^ for Ag NPs and 0.36 mg/g^−1^ for ionic silver (Ag^+^). The unbounded electron of nitrogen atoms would be available for the coordination of Ag^+^. Thus, the immobilized DMC sequesters silver despite its form. Ag NPs and ionic silver (AgNO_3_) are mostly recovered. A support composed of positively charged quaternary trimethylated amines (TMC) was also used to verify if it would be more selective for Ag NPs. The ionic silver in presence of immobilized TMC (DQ of 47.6 %) was sequestrated at 28.0 % (line 8) and 23.0 % of Ag NPs for immobilized TMC (line 9). The decrease of sequestration would be explained by the steric hindrance around the cationic charge of the polymer.

**Table 1:** Percentage of sequestration of Ag NPs, Ag^+^ and Ag_2_S NPs by immobilized DMC at different conditions

Line	Time of sequestration (h)	Parameters	Percentage of sequestration (%)^a^
1	0.5	25 °C	59.9 ± 4.1
2	2	25 °C	77.7 ± 3.9
3	4	25 °C	84.2 ± 5.8
4	4	2 °C	3.5 ± 0.2
5	4	40 °C	26.9 ± 0.5
6	4	1.34 mg L^−1^ of Ag^+^	100.0 ± 0.0
7	4	4.25 mg L^−1^ of Ag^+^	84.2 ± 4.8
8	4	4.25 mg L^−1^ of Ag^+^; with immobilized TMC	28.0 ± 8.4
9	4	With immobilized TMC	23.0 ± 1.4
10	4	+16.0 mg L^−1^ of Ag_2_S	24.1 ± 9.1

Ag NPs (120 µg L^−1^) were added by default excepted in cases where the source of silver is mentioned. Ag^+^ was added as silver nitrate

^a^Average ± SD

In an environment with high concentrations of sulfur like municipal wastewater, Ag NPs can also be transformed into Ag_2_S [21]. The Ag_2_S nanoparticles of size of 77.1 ± 56.8 nm were synthesized from l-cysteine and silver nitrate. The immobilized DMC sequestrated 24.1 % of Ag_2_S NPs (line 10) corresponding to a sorption capacity of 0.39 mg g^−1^. The zeta potential was used to quantify the nanoparticle charge and provide information on electrostatic interactions (Table 2). The zeta potential of the Ag NPs was −7.4 mV (line 11) while the zeta potential of Ag_2_S NPs was −6.1 mV. With a zeta potential being less negative, Ag_2_S NPs would be more difficultly adsorbed on the immobilized DMC (line 8).

**Table 2:** Zeta potential of Ag NPs (120 µg L^−1^) by addition of electrolytes

Line	Parameters	Zeta potential of Ag NPs (mV)^a^
11	Nanopure water	−7.4 ± 1.2
12	20 mg L^−1^ NaSH	−49.3 ± 0.8
13	35.2 mg L^−1^ SDS	−9.0 ± 1.3
14	20 mg L^−1^ NaSH; 8.8 mg L^−1^ SDS	−58.2 ± 3.1

^a^Average ± SD

Table 3 shows the percentage of sequestration of Ag NPs, by the immobilized DMC, increases with addition of hydrosulfide. The hydrosulfide concentrations correspond to the minimum amounts of sulfur found in wastewater according to Hurse and Abeydeera [22]. Hydrosulfide ions can strongly coordinate silver because they modify the electronic environment and creates strong covalent bonds [23]. By coordinating the surface of Ag NPs, the particle becomes strongly negative. Indeed, the zeta potential of Ag NPs was −7.4 mV (line 11) while the zeta potential of Ag NPs with hydrosulfide ions was −49.3 mV (line 12). This strong negative charge promotes electrostatic interactions with the cationic immobilized DMC.

**Table 3:** Percentage of sequestration of Ag NPs (120 µg L^−1^) by immobilized DMC with addition of NaSH

Line	NaSH concentration (mg L^−1^)	Molar ratio NaSH/Ag NP	Percentage of sequestration (%)^a^
15	0	0	84.2 ± 4.8
16	20	0.2	99.5 ± 0.2
17	100	1.0	100.0 ± 0.0
18	200	2.0	99.6 ± 0.6

^a^Average ± SD

Sodium dodecyl sulfate (SDS) and cetyl alcohol are surfactants commonly used in consumer products, which are found in municipal wastewater. Surfactants could affect Ag NPs properties and their interactions with immobilized DMC [24]. SDS concentrations used in experiments were the upper and lower limits found in wastewater influents in the USA according to Knepper and coworkers [25], whereas the cetyl alcohol concentration is limited by the solubility. In the presence of SDS, an anionic surfactant (Table 4, lines 19–21), the percentage of sequestration of Ag NPs decreases to around 20 %. In the presence of sodium *N*-lauroylsarcosinate (SLS), another anionic surfactant (line 22), the percentage of sequestration decreases to 2.7 %. The zeta potential of Ag NPs in water was −7.4 mV (line 11) while the zeta potential with addition of SDS was −9.0 mV (line 13). The charge on the surface does not change within precision. Surfactants, due to their partial charge (SLS -1/2 and SDS -1/3 per oxygen atom), would replace the citrate ion and would increase the hydrophobicity of Ag NPs. The partial charge of SLS being greater than SDS would coordinate more Ag NPs and replace more the citrate ion, hence the lower sequestration by addition of SLS. Highly hydrophobic species could reduce sequestration. In the presence of cetyl alcohol, a non-ionic surfactant (lines 25–26), the percentage of sequestration became at around 4 %. The same reduction due to hydrophobicity occurs with cetyl alcohol. Ag NP behavior in wastewater would be changed. In the presence of both SDS and sulfide, the DMC sequestered 6 % of Ag NP (line 23). In this case, the zeta potential was −58.2 mV (line 14). The particles are strongly negative as well as being very hydrophobic that prevents sequestration by DMC.

**Table 4:** Percentage of sequestration of Ag NPs by immobilized DMC at differents conditions after 4 h

Line	Parameters	Percentage of sequestration (%)^a^
19	3.6 µg L^−1^ SDS	27.0 ± 1.4
20	8.8 mg L^−1^ SDS	21.6 ± 7.9
21	35.2 mg L^−1^ SDS	23.5 ± 3.3
22	11.8 mg L^−1^ SLS	2.7 ± 0.6
23	8.8 mg L^−1^ SDS; 20 mg L^−1^ NaSH	6.0 ± 0.8
24	20 mg L^−1^ NaSH; 8.8 mg L^−1^ SDS	6.4 ± 0.6
25	0.335 µg L^−1^ cetyl alcohol	2.4 ± 0.5
26	1.34 µg L^−1^ cetyl alcohol	5.9 ± 0.1

The concentration of Ag NPs was 120 μg L^-1^

^a^Average ± SD

The sequestration percentage reached very high values as high as 99–100 % (Table 3, lines 16–18) for solution containing sodium hydrosulfide and decreased to 90.7 % (line 27) by addition of 10 % municipal wastewater. Municipal wastewater contains compounds like sulfur and organic matter leading to a decrease of sequestration (lines 27–29). A solution composed of 50 % wastewater gave a sequestration of 84.6 % (line 28) while a solution of 97 % wastewater had a percentage of sequestration of 51.7 % by immobilized DMC (line 29). In the absence of suspended matter—municipal wastewater previously filtered through GF/F 0.7 μm—the percentage of sequestration was 27.4 % (line 30). The organic matter is known to form complexes with silver [23]. The presence of humic substances stabilizes Ag NPs by covering them that reduced agglomeration or sedimentation [26]. The lower electrostatic charge would decrease interaction with the cationic immobilized DMC as observed in the presence of organic matter (Table 5).

**Table 5:** Percentage of sequestration of Ag NPs (120 µg L^−1^) by immobilized DMC in wastewater after 4 h

Line	Composition of aqueous solutions	Percentage of sequestration (%)^a^
27	10 % wastewater	90.7 ± 0.2
28	50 % wastewater	84.6 ± 7.6
29	97 % wastewater	51.7 ± 6.7
30	97 % wastewater filtered GF/F 0.7 μm	27.4 ± 5.8

^a^Average ± SD

### Characterization of immobilized DMC after sequestration of Ag NP

**Fig. 3:**
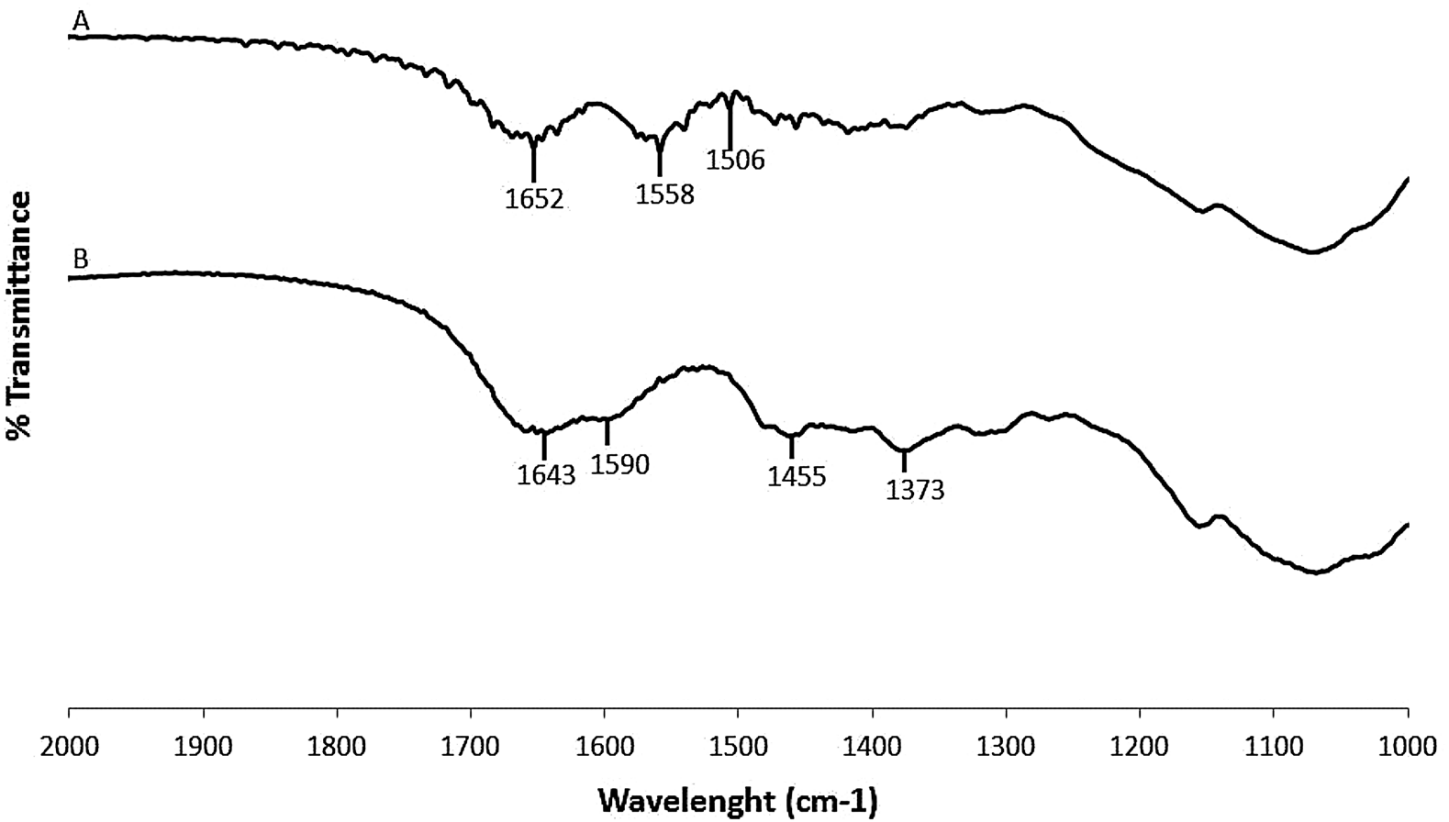
Infrared spectra of immobilized DMC after (*A*) and before (*B*) Ag NP sequestration

Figure 3 shows the infrared spectra of immobilized DMC before and after sequestration of citrate coated Ag NPs. Figure 3a (after sequestration) shows a band at 1558 cm^−1^ associated to asymmetric carboxylate stretching band of citrate carbonyl on Ag NPs [27] while Fig. 3b (before sequestration) does not have any band in this region. The C = O stretching in Fig. 3a (after sequestration) indicates that Ag NPs were sequestered by the immobilized DMC.

**Fig. 4:**
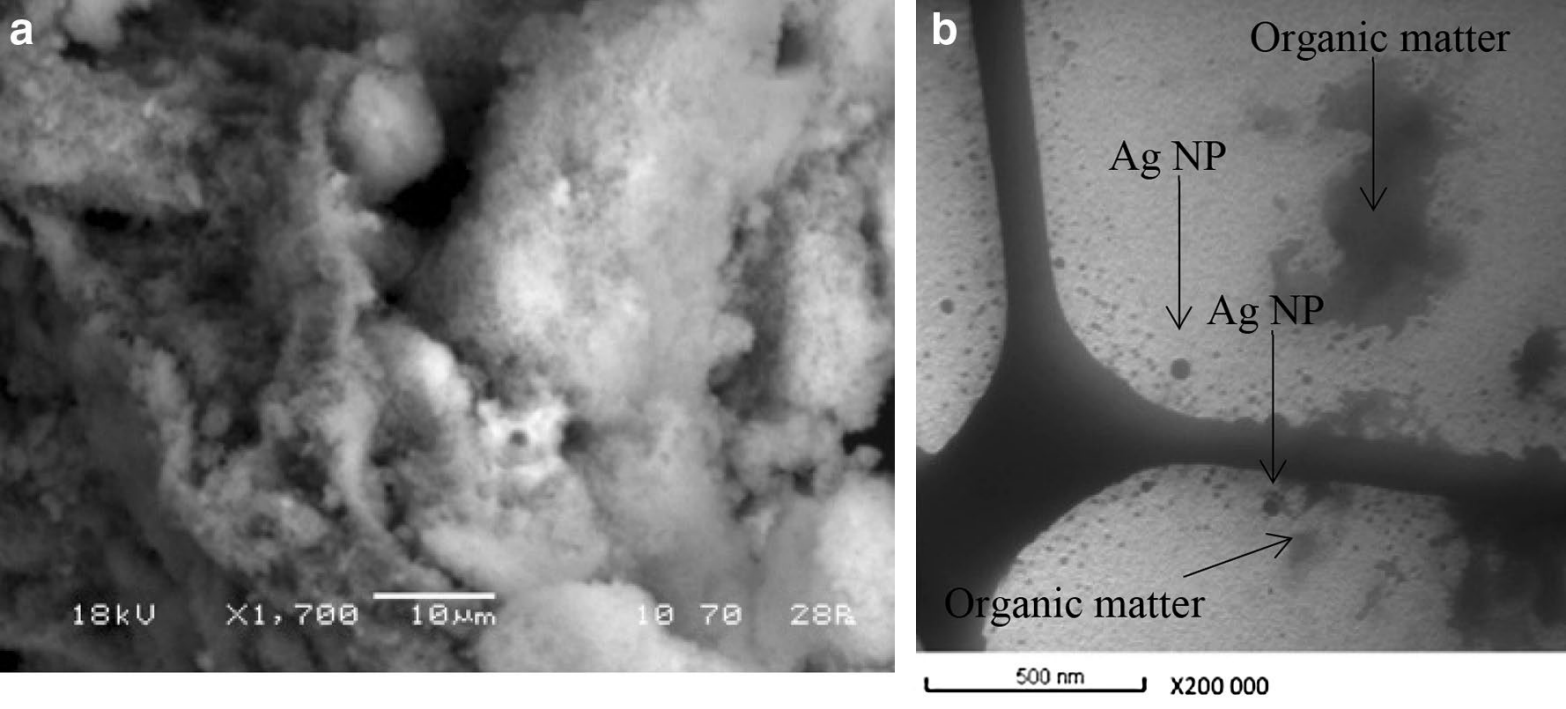
**a** Scanning electron microscope (*SEM*) image of immobilized DMC and **b** transmission electron microscope (*TEM*) image of Ag NPs after sequestration

SEM allows visualizing certain characteristics like the size and morphology. Figure 4 shows SEM image (Fig. 4a) of the immobilized DMC after Ag NP sequestration in water. There are no observable structural differences in SEM between the silica (not shown) and immobilized DMC. Thus, the silica would have a homogeneous covering of DMC, which is coherent with the IR spectrum (Fig. 1). SEM images show that the immobilized DMC is porous. In Fig. 4b, the black dots on TEM image represent Ag NPs of 20 nm size while the light gray shape without distinct outline would be organic matter (DMC or citrate).

**Fig. 5:**
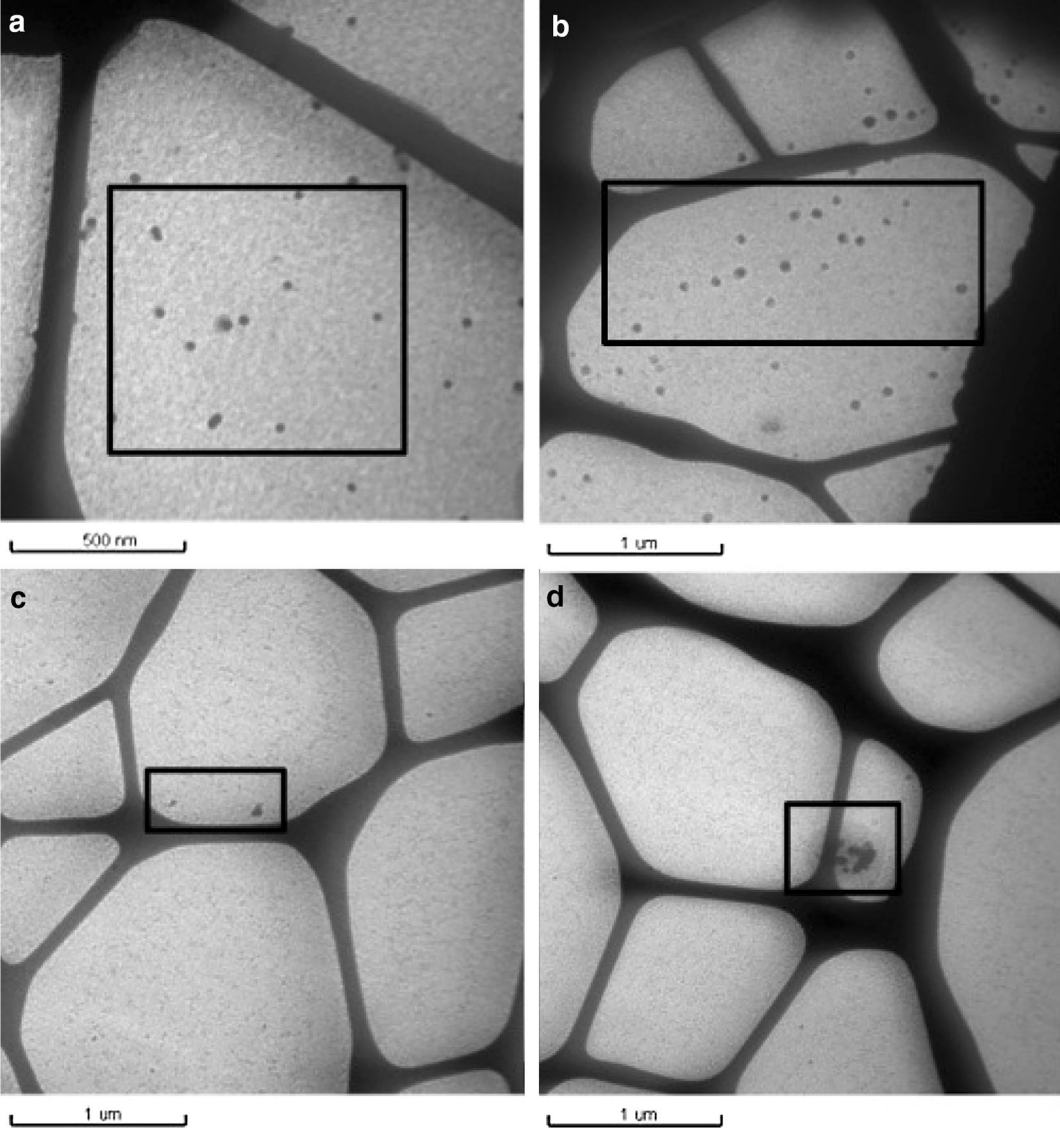
TEM images of **a** Ag NPs citrate; **b** Ag NPs citrate with NaSH in molar ratio NaSH/Ag NP 1:5 after sequestration; **c**, **d** Ag NP citrate with NaSH in molar ratio NaSH/Ag NP 1:5

**Fig. 6:**
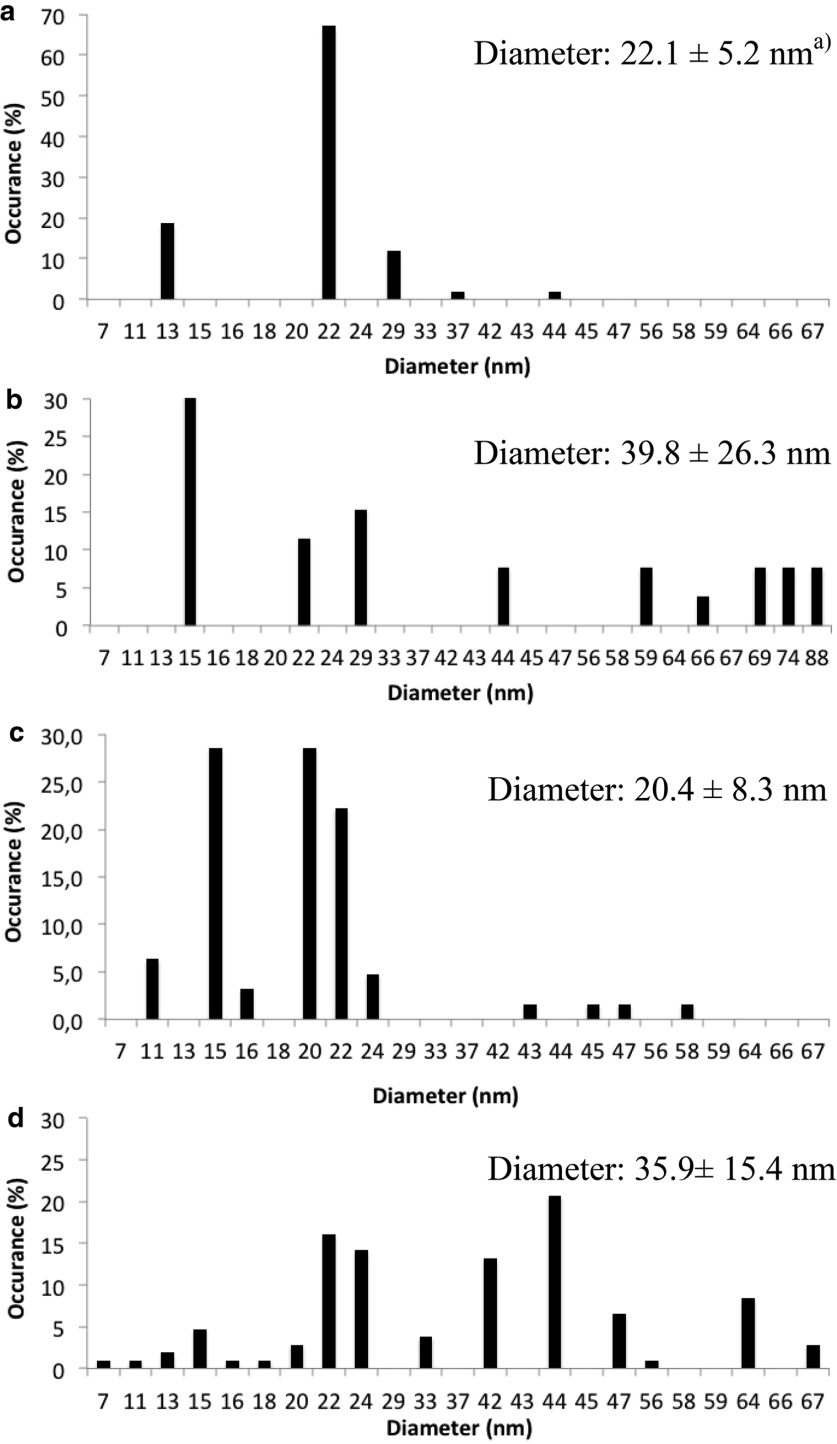
Particle size distribution (n = 100) from TEM images of **a** Ag NPs before sequestration; **b** Ag NPs after sequestration; **c** Ag NPs with NaSH before sequestration; **d** Ag NPs with NaSH after sequestration

The average diameter of Ag NPs and their size distribution can be determined by TEM. In the stock solution, citrate-coated Ag NPs do not agglomerate (Fig. 5a), Ag NPs are monodisperse with an average diameter of 22.1 nm (Fig. 6a). Adding NaSH, a part of Ag NPs agglomerates while the other part remains in monomeric form (Fig. 5c, d) with an average diameter of 20.4 nm (Fig. 6c). After sequestration in nanopure water (Fig. 4b) or NaSH solution (Fig. 5b), Ag NPs appeared with defined sizes without agglomeration. However they are polydispersed with sizes of 15, 22–29, 44 and 59–88 nm, resulting in an average diameter of 39.8 nm (Fig. 6b). After sequestration in NaSH solution, the range was mainly between 20 and 24 nm and 42–44 nm, with an average diameter of 35.9 nm (Fig. 6d). During sequestration, the DMC counterion (acetate ion) could exchange with the citrate ion. Thus, Ag NPs would be less stable and will agglomerate.

### Effect of temperature on sequestration

By varying the temperature during sequestration, it was possible to determine the activation energy from the Arrhenius relationship. A plot of 1/T according to the natural logarithm of the first order rate constant is performed. The slope of the line corresponds to the activation energy divided by the gas constant (8.314 J K^−1^ mol^−1^). The activation energy was 803 J mol^−1^. The immobilized DMC sequesters 3.5 % at 275 K, 84.2 % at 298 K and 26.9 % at 313 K (Table 1, lines 3–5). Sequestration was largely affected by temperature where the best sequestration was obtained at 25 °C. At 2 °C, the activation energy is not completely attained and a low amount of Ag NP is sequestered by the immobilized DMC. This energy is achieved at room temperature. Thus, environmental samples could be easily handled. At 40 °C, the activation energy is reached and environmental temperature increases the molecular motion.

The increase of temperature in the reaction medium would result in competitive reactions explaining the low percentage of sequestration. When modifying the order of addition between NaSH and SDS, the percentage of sequestration of Ag NPs is similar, 6.0 % when SDS (Table 4, line 23) is added first compared to 6.4 % when NaSH (line 24) is added first. These observations indicate that the process is reversible and that there is competition between anions.

## Experimental

### General information

SiliaBond^®^ propyl bromide (particle size 40–63 µm, loading 1.69 mmol/g, specific surface area 470–530 m^2^ g^−1^), chitosan (viscosity <20 mPa s (cP), degree of deacetylation >95 % from shrimp exoskeletons, *Pandalus borealis*) and silver nanoparticles (20 nm, 0.02 mg/mL) coated with citrate were purchased respectively from Silicycle (Quebec), Primex (Iceland) and TedPella (USA). All other reagents were bought from Aldrich except sodium *N*-lauroyl sarcosinate (ICN biomedicals). Sodium dodecyl sulfate (SDS) was reagent plus grade. Concentrated nitric acid (≥69 % v/v) and hydrogen peroxide (≥30 % v/v) were ultrapure grade whereas other reagents were ACS grade. *N*,*N*–dimethylchitosan (DMC) was synthesized according to literature [28]. Nanopure water was obtained from a Barnstead nanopure infinity ultrapure water system. Ionic silver comes from AgNO_3_. All materials were washed with nitric acid and rinsed with nanopure water before use. Municipal wastewater was collected on June 18, 2013 as a 24 h-composite sample from aerated lagoons at Rimouski-Est station (Quebec, Canada). The sample was stored at −20 °C. Municipal wastewater had 0.61 g L^−1^ of total matter and 0.46 g L^−1^ of dissolved matter. The microwave heating was realized with a Mars microwave system from CEM Corporation using MarsXpress™ close-vessels. Infrared and Raman spectra were recorded on a Thermo scientific Nicolet iS10 spectrometer with Smart Omni transmission in KBr pellets and on a Thermo scientific DXR Raman Microscope directly on solid, respectively. Elemental analyses were determined using analyzer Costech instruments elemental combustion system 4100. NMR spectra were performed using an Avance III HD 600 MHz NMR from Bruker by NanoQAM (Université du Québec à Montréal). UV–visible spectra were recorded on a Cary 100 Bio UV–visible spectrophotometer from Varian. ICP–MS measurements were achieved on an Agilent 7500c spectrometer octopole reaction system using argon plasma at 7000 K, autosampler ASX-520 Cetac and software ChemStation v.3.04. Analyses from MP-AES were achieved on an Agilent Technologies 4200 MP-AES with a nitrogen generator, autosampler ASX-520 Cetac and MP Expert software version 1.5.0.6545. Zeta potentials were measured by Malvern zetasizer nano ZS with Malvern Zetasizer software version 7.11. Solutions were placed in disposable capillary cells (DTS1070) of Malvern which were washed with nanopure water, nitric acid 10 % v/v, nanopure water and ethanol. A single measurement with zetasizer had 100 runs in manual mode, the zetasizer took three measurements with a delay of 45 s. Zeta potentials of Ag NPs were measured in nanopure water excepted when presence of salts is mentioned. Transmission electron microscopy (TEM) was recorded on a Delong Instruments model LVEM5. Before TEM analyses, dried supports were ground in an agate mortar and then suspended in dry ethanol. A few drops of solution were placed on a copper grid of 400 mesh covered with a hexagonal carbon film provided by Ted Pella Inc. (Redding, CA). SEM microscope was a JEOL JSM-6460 LV scanning electron microscope. Dried supports were placed on a carbon tape and placed on the sample holder. The uncertainty of zeta potential measurements was estimated using the standard deviation between three data collections. The uncertainty on percentage of sequestration comes from the standard deviation between two independent sequestrations.

### General procedure for the preparation of immobilized DMC on modified silica (example for polymer/silica ratio 1:1)

A suspension containing DMC (0.30 g), sodium carbonate (0.90 g) and SiliaBond^®^ propyl bromide (0.32 g) was prepared in a mixture of methanol/water (8 mL, 1:9 v/v). In MarsXpress™ close-vessels, the suspension was heated by microwave at 100 °C during 5 min and the temperature was maintained at 100 °C during 15 min using a maximum power of 1600 W. The solution was allowed to reach room temperature (rt). The solid was filtered and suspended in a 1 % (v/v) aqueous acetic acid solution (50 mL) during 15–30 min. The solid was filtered and dried at normal atmosphere. A white solid was obtained (0.33 g). The solid was ground to a size of 250 μm. IR υ (cm^−1^) 3430 (OH), 2900 (CH), 1558 (CH_2_ def), 1462 (CH_3_ def), 1380–1265 (C–N), 1110–1090 (C–O pyranosyl). Raman υ (cm^−1^) 634, 562 (C–Br).

### General procedure for the N-methylation of immobilized DMC on modified silica (polymer/silica ratio 1:1)

A suspension containing immobilized DMC (0.30 g), sodium carbonate (0.90 g) in a mixture of methanol/water (8 mL, 1:9 v/v) and iodomethane (3 mL). In MarsXpress™ close-vessels, the suspension was heated by microwave at 100 °C during 5 min and the temperature was maintained at 100 °C during 15 min using a maximum power of 1600 W. The solution was allowed to reach rt. The solid was then filtered and dried under normal atmosphere. The yield is quantitative. Degree of quaternization (DQ) of TMC in the protonated form was obtained by comparing the integrals of N(CH_3_)_3_^+^ (3.3 ppm), N(CH_3_)_2_ (3.0 ppm) and CH_3_CO (2.1 ppm) peaks from the ^1^H NMR spectrum in D_2_O. DQ of TMC was 47.6 % from the following equation.

$${\text{DQ = }}\frac{{{\text{N(CH}}_{3} )_{3}^{ + } /9}}{{({\text{N(CH}}_{3} ) _{3}^{ + } / 9 ) {\text{ + (N(CH}}_{3} )_{2} / 6 ) {\text{ + (CH}}_{3} {\text{CO}}/ 3 )}} \times 100\,\%$$

A suspension containing TMC (0.30 g), sodium carbonate (0.90 g) and SiliaBond^®^ propyl bromide (0.32 g) in a mixture of methanol/water (8 mL, 1:9 v/v). In MarsXpress™ close-vessels, the suspension was heated by microwave at 100 °C during 5 min and the temperature was maintained at 100 °C during 15 min using a maximum power of 1600 W. The solution was allowed to reach rt. The solid was then filtered and dried under normal atmosphere. The solid was ground to a size of 250 μm.

### Procedure for formation of Ag_2_S nanoparticles

The synthesis method of Ag_2_S nanoparticles was adapted from Xiang and coworkers [29] and Brelle and coworkers [30]. Silver nitrate (68 µmol, 11.5 mg) was added to a stirred solution of l-cysteine (68 µmol, 8.2 mg) in 10 mL ethanol. After 15 min, the solution was transferred into a 15 mL Teflon tube. The tube was placed in 120 mL high pressure reactor from Parr Instrument. The reactor was heated at 180 °C during 10 h after that it was allowed to reach rt. The resulting precipitate was centrifuged at 3000 rpm during 10 min and washed using nanopure water and absolute ethanol several times. The dark precipitate was dried at 60 °C during 6 h. The black, dried precipitate was then put into a tube with ethanol and placed in a bath sonicator for 5 min. The solution was decanted for 1 h. The suspension was recovered and evaporated. The Ag_2_S NP mean size of 77.1 ± 56.8 nm was determined by TEM.

### General procedure of sequestration of silver nanoparticles

Ag NP solution was prepared by dilution (factor 33×) of the commercial stock solution. In a Falcon tube (15 mL), the Ag NP solution (10 mL) and immobilized DMC on silica (0.100 g) were stirred with a magnetic bar during 4 h. The sequestrations were carried out in duplicate. The suspension was then centrifuged at 1000×*g* for 5 min. The supernatant was first collected and the residual solid was filtered (Whatman cellulose filter papers grade 2) and dried under normal atmosphere. The samples were placed in the dark at 4 °C until further analysis. IR of immobilized DMC after sequestration υ (cm^−1^) 3430 (OH), 2900 (CH), 1651 (C = O in COOH), 1557 (CH_2_ def), 1110–1090 (C–O pyranosyl).

### Sample preparation prior to ICP–MS and MP-AES analyses

Dried support (0.100 g), concentrated nitric acid (6 mL) and hydrogen peroxide (1 mL) were mixed in open flasks until the complete gas evolution during at least 2 h. The flasks were closed and heated to 70 °C in a hot bath for an additional 2 h. The supernatant (2 mL) was digested in the same way than for the support except that 4 mL of nitric acid was used. The samples were stored in dark at 4 °C until ICP-MS or MP-AES analyses. All analyses were performed with the ICP-MS except analyses with TMC immobilized, Ag_2_S, Ag NPs at 2 and 40 °C, NaSH with SDS and SLS that have been made by MP-AES. The detection of ^107^Ag was used to measure the total silver in ICP–MS. The limit of detection for silver by ICP–MS was 0.04 µg L^−1^.

## Conclusions

The synthesis of immobilized DMC on silica meets some principles of green chemistry [31, 32], for example, by using renewable products and microwave-assisted heating. Ag NPs are retained in the immobilized DMC by electrostatic interactions. When Ag NPs are highly negatively charged, for example by addition of NaSH, interactions are stronger and thus the sorption efficiency increases. Sequestrations are also highly dependent of hydrophobicity of Ag NPs coming from surfactants or organic matter that would decrease the electrostatic interactions with Ag NPs and lower the sorption efficiency. In a more complex environment, many factors likely influence the interactions between Ag NPs and immobilized DMC resulting in lower sorption capacity.

Immobilized polysaccharides as chitosan derivatives could serve as a promising approach for retrieving or removing emerging pollutants and heavy metals due to the chelation of the nitrogen atom. The immobilized DMC can be used on large volume samples and at low metallic pollutant concentrations. Sequestration optimizations should be carried out to increase selectivity and sensitivity of the method for potential number of uses.

## Additional file

10.1186/s13065-016-0198-4 Infrared and Raman spectra of immobilized DMC, variation of N/C ratio, UV-visible spectra of Ag NP solutions.
