# Supplementary material for: New method for sequestration of silver nanoparticles in aqueous media: in route toward municipal wastewater
Source: Chem Cent J. 2016 Aug 26;10(1):54. doi: 10.1186/s13065-016-0198-4 (PMC5002176; doi:10.1186/s13065-016-0198-4)
Supplement: Supplementary file 1 — 10.1186/s13065-016-0198-4 Infrared and Raman spectra of immobilized DMC, variation of N/C ratio, UV-visible spectra of Ag NP solutions. [file 13065_2016_198_MOESM1_ESM.docx]

**New method for sequestration of silver nanoparticles in aqueous media: In route toward municipal wastewater**

Supporting information

Marie-Laine Roy (marie-laine_roy@hotmail.com)

Christian Gagnon (christian.gagnon@ec.gc.ca)

Jonathan Gagnon (jonathan_gagnon@uqar.ca)


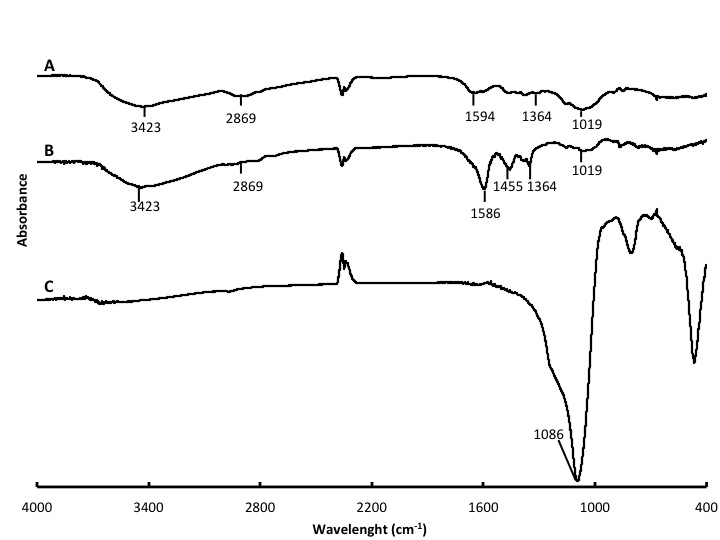


Figure S1. Infrared spectra of (A) immobilized DMC after washing; (B) DMC; (C) silica propyl bromide


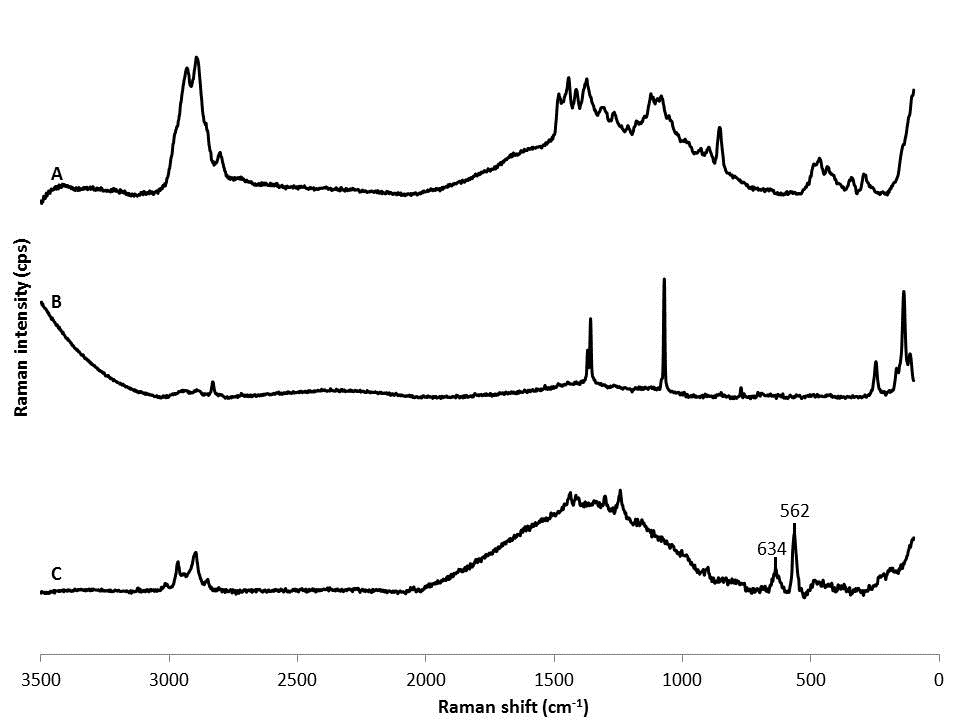


Figure S2. Raman spectra of (A) immobilized DMC; (B) DMC; (C) silica propyl bromide


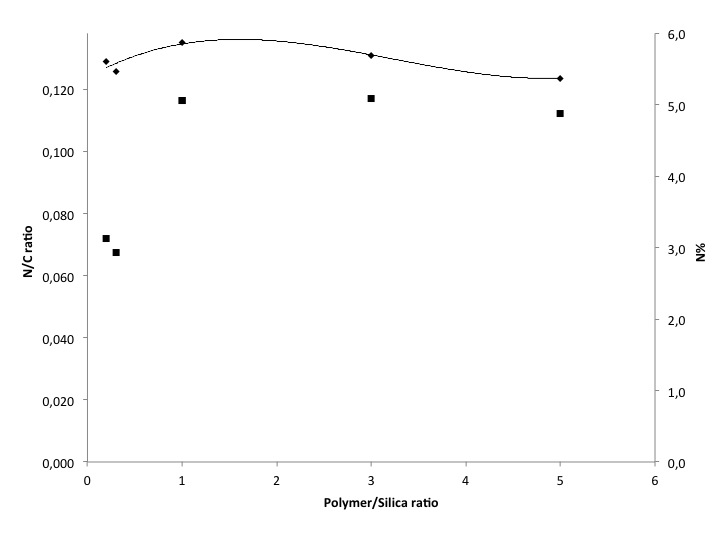
Figure S3. Nitrogen/carbon ratio (♦) and nitrogen percentage (⏹) of immobilized DMC on modified silica with the variation of the polymer/silica ratio


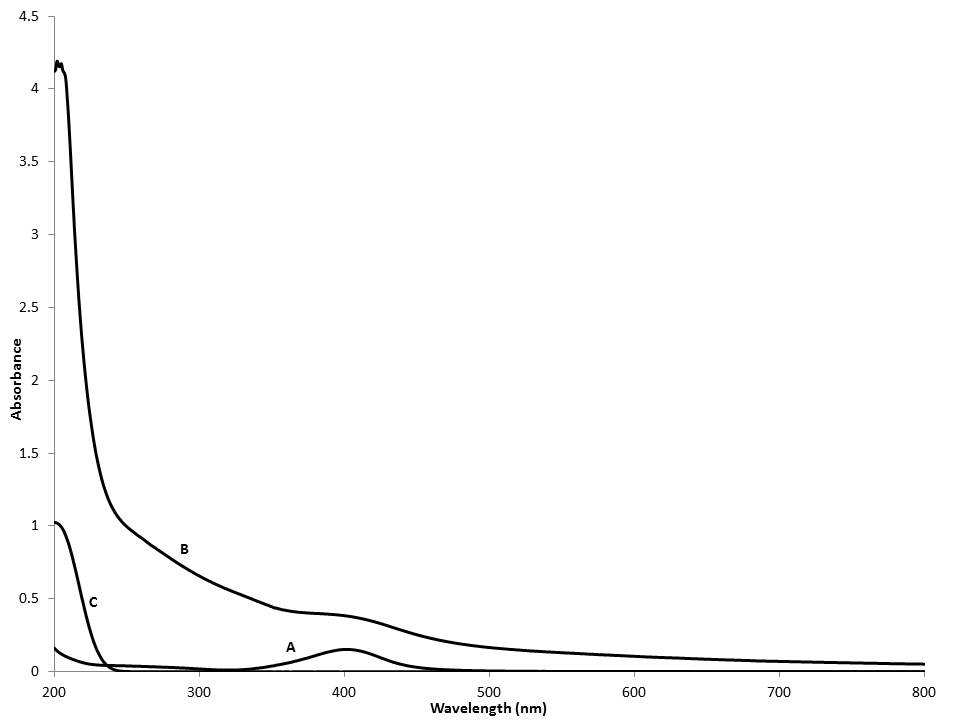


Figure S4. UV-visible spectra of a solution of A) citrate Ag NPs; B) supernatant after sequestration; C) acetic acid
